# Biosecurity in an age of open science

**DOI:** 10.1371/journal.pbio.3001600

**Published:** 2022-04-14

**Authors:** James Andrew Smith, Jonas B. Sandbrink

**Affiliations:** 1 Botnar Research Centre and Centre for Statistics in Medicine, Nuffield Department of Orthopaedics, Rheumatology and Musculoskeletal Sciences, University of Oxford, Oxford, United Kingdom; 2 National Institute for Health Research Oxford Biomedical Research Centre, John Radcliffe Hospital, Oxford, United Kingdom; 3 Nuffield Department of Medicine, University of Oxford, Oxford, United Kingdom; 4 Future of Humanity Institute, University of Oxford, Oxford, United Kingdom

## Abstract

The risk of accidental or deliberate misuse of biological research is increasing as biotechnology advances. As open science becomes widespread, we must consider its impact on those risks and develop solutions that ensure security while facilitating scientific progress. Here, we examine the interaction between open science practices and biosecurity and biosafety to identify risks and opportunities for risk mitigation. Increasing the availability of computational tools, datasets, and protocols could increase risks from research with misuse potential. For instance, in the context of viral engineering, open code, data, and materials may increase the risk of release of enhanced pathogens. For this dangerous subset of research, both open science and biosecurity goals may be achieved by using access-controlled repositories or application programming interfaces. While preprints accelerate dissemination of findings, their increased use could challenge strategies for risk mitigation at the publication stage. This highlights the importance of oversight earlier in the research lifecycle. Preregistration of research, a practice promoted by the open science community, provides an opportunity for achieving biosecurity risk assessment at the conception of research. Open science and biosecurity experts have an important role to play in enabling responsible research with maximal societal benefit.

## Abstract 2

The risk of biotechnology being misused is growing and may even be increased by moves towards open science. How can we ensure that the goals of both open science and biosecurity are met?

## Introduction

Open science aims to increase the reliability and efficiency of scientific research [1,2]. Despite the importance of work to improve scientific practice, increased openness may increase the chance of deliberate or accidental misuse of research. These concerns are particularly salient for biological research on pathogen synthesis and engineering.

Here, we examine how certain open science practices interface with risks arising from the potential misuse of biological research. For the majority of biological research, increased openness is likely to improve our ability to deal with biological threats through improving the efficiency and reliability of science. However, the subset of research in which biological risks may be increased should not be ignored, and tools and systems to encourage and facilitate responsible access to scientific findings must be available. Collaboration between the open science community and the biosecurity and biosafety communities may be mutually beneficial and will allow more consideration of the potential risks and opportunities associated with open science. Addressing risks and opportunities raised in this paper should help to reduce threats that could undermine the significant progress made in open science to date and have catastrophic consequences for society.

## Risks from biological research

Natural pandemics have posed major threats to human populations throughout history. In recent decades, research in immunology, virology, and other biomedical domains has greatly improved global pandemic preparedness. However, some life science research has the potential to be misused. Misuse risks can be classified into biosafety risks, concerning accidental exposure and release, and biosecurity risks, concerning deliberate misuse [3]. As biotechnology grows more powerful and accessible [4], risks from accidental or deliberate misuse of research may increase.

There are numerous examples of high-profile biosafety incidents [5]. The 1977 H1N1 influenza epidemic likely resulted from vaccine trials in the Soviet Union, or accidental release from a laboratory, reintroducing a strain that was circulating in the 1950s [6,7]. In 2015, the United States realised that it had distributed live anthrax, as opposed to the intended inactivated anthrax, in 575 shipments to 8 countries over a decade [8]. Laboratory accidents involving dangerous pathogens happen frequently and, given the leading cause of such accidents is human error, are difficult to mitigate completely even in high-level biocontainment facilities [9]. For instance, in 2003 and 2004, incidents in 3 different labs researching or containing SARS-CoV-1 lead to a total of 6 lab-acquired infections, which could have sparked further epidemics [10].

Deliberate release of pathogens may take place in the context of biological warfare or terrorism. A historic precedent for the intentional misuse of biological agents is set by the Soviet Union’s extensive biological weapons program that involved the synthesis and enhancement of plague, smallpox, and other agents [11]. There have also been terrorist incidents such as the 2001 US anthrax attacks, likely conducted by a single perpetrator with training and access to biological research materials, showing that greater access to materials and methods can have tragic consequences when abused [12].

Certain life sciences research may be misused and increase the risk from deliberate biological events. For example, though advances in viral engineering may be important in areas like vaccine design and cancer therapy, they could be applied to engineer pathogens with increased virulence or transmissibility. Deliberate release of such pathogens could result in a pandemic of unprecedented severity. Research with the greatest misuse potential has been labelled dual-use research of concern (DURC), defined by the National Institutes of Health in the US as “life sciences research that, based on current understanding, can be reasonably anticipated to provide knowledge, information, products or technologies that could be directly misapplied to pose a significant threat with broad potential consequences to public health and safety” [13]. According to the Global Health Security Index, only 1% of countries have appropriate oversight for potential dual-use life science research with especially dangerous pathogens [14]. The few existing frameworks are limited in scope; for instance, the US DURC policies only apply to research on 15 select agents and toxins at federally funded institutions [15,16].

That certain information may cause harm and should not be publicly accessible has long been accepted in nuclear physics. In 1946, the US Atomic Energy act turned all information on nuclear weapons into “restricted data” until formally declassified [17]. Today, information hazards in the life sciences, i.e., knowledge and insights that can cause harm, are frequently associated with greater dual-use potential than physical materials [18,19]. Advances in molecular biology, including DNA synthesis and gene editing, are democratising science and lowering the barrier to the synthesis and engineering of biological agents [20]. As biotechnology advances, we need to acknowledge that biological research, similarly to nuclear physics, may uncover information with security implications and consider the possibility that not all information should be made publicly available.

## Open science and risks from biological research

“Open science” has been advanced [21–23] in part to address widespread problems identified across the sciences [24]; however, there is little consensus on what the term, or related terms such as “reproducibility”, mean [25–27]. We consider open science to be a set of practices that aim to improve the reliability and efficiency of scientific research [23,28] that are generally characterised by increased transparency. We consider that open science achieves its aims through 3 instrumental mechanisms: accessibility, verification, and reuse. By accessibility, we mean making research outputs widely, and usually publicly, available. By verification, we mean the ability to review and critique aspects of the research to establish that what is described corresponds to what was done. An example of this is computational reproducibility, i.e., ensuring that the data and code achieve the claimed results. By reuse, we mean the ability to use research outputs for the same or another purpose, such as conducting a replication study, validation study, or secondary analysis. Accessibility in many cases facilitates efforts to verify and reuse research results.

Open science may contribute to mitigating biosecurity and biosafety risks. Reliable and efficient science is important for effectively preventing and responding to pandemic risks: for example, in developing drugs, vaccines, and diagnostics, and implementing effective public health responses. Preprints may have played an important role in scientific and public engagement with COVID-19 research [29]. Research excellence and ethical research conduct, both encouraged by open science, are pillars of responsible life science research for global health security [30]. The move towards open science has involved a cultural shift related to conduct and sharing and research; a similar cultural shift may be required to encourage responsible research conduct and sharing to protect the life sciences from misuse. Open science may therefore represent a useful case study [31].

**Fig 1:**
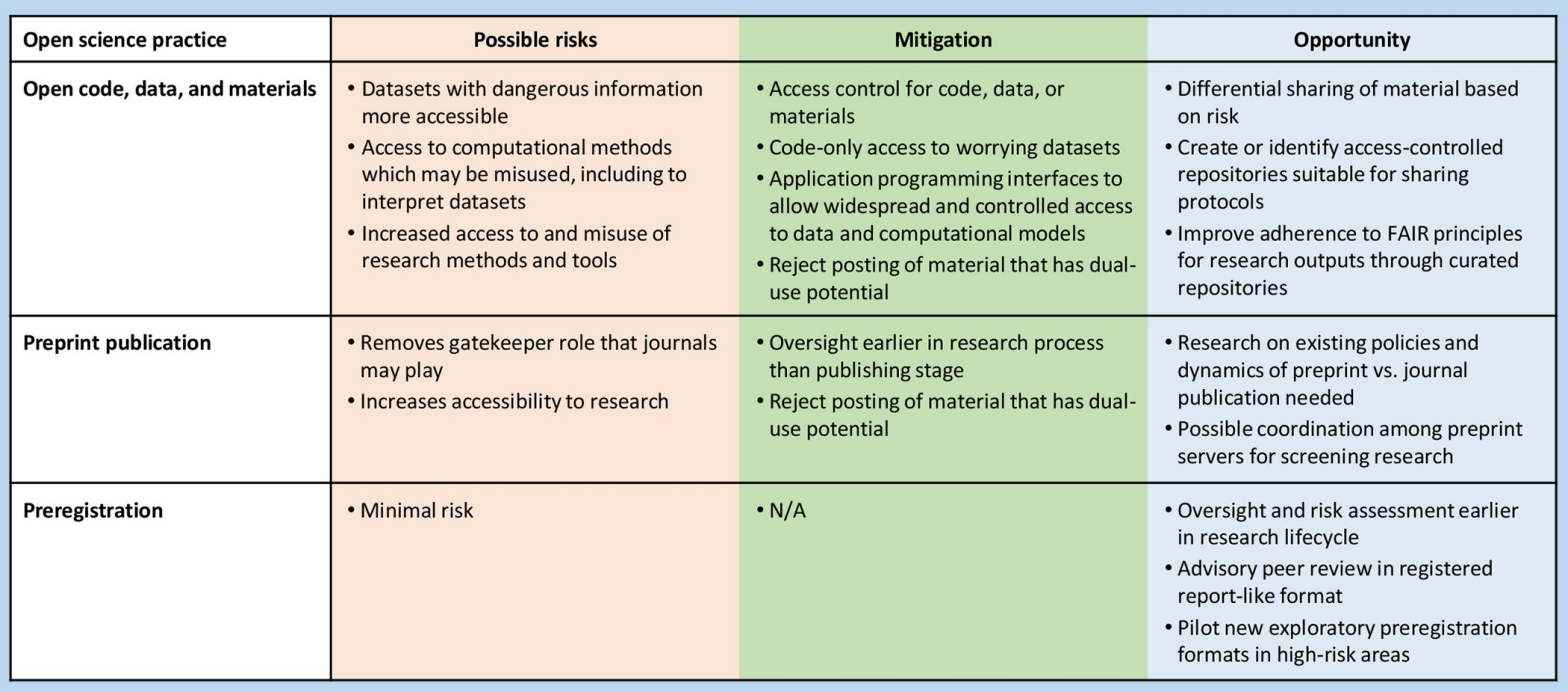
Summary of risks arising from open science practices, strategies to mitigate these risks, and opportunities to improve biosecurity and biosafety.

As we highlight, however, there are instances where open science may exacerbate biosecurity and biosafety risks. Addressing such risks will inevitably reduce the ability to verify, reuse, or access research. However, this need not necessarily reduce reliability and efficiency. When typical open science practices seem inadvisable, we provide tentative suggestions for how reliability and efficiency can still be improved. We focus on 3 practices that appear relevant to biological risks: open code, data, and materials; preprint publication; and preregistration (Fig 1). These reflect, for example, the 3 options provided in the “Conduct your own open science” section on the Centre for Open Science home page: https://www.cos.io/.

## Open code, data, and materials: A challenge for mitigating misuse

Sharing code and data allows the research community to reproduce and check analytical findings [32,33] and facilitates reuse. Code is typically shared via repositories such as the Open Science Framework, GitHub, Zenodo, or in supplementary files accompanying a published article. Data may be shared similarly, though discipline-specific repositories [34] are also common. By open materials, we mean detailed, recipe-like explanations (such as written instructions or videos) on how to do certain procedures (we limit discussion to digital rather than physical materials because physical materials are already more highly regulated and different considerations apply to them. However, physical materials are included in some definitions of open materials) [35]. Such materials can be shared in supplementary files, dedicated repositories like protocols.io or bio-protocol.org, general purpose repositories like Open Science Framework, or as stand-alone journal articles (e.g., [36]).

Code could be used directly or adapted to achieve nefarious goals [37,38]. Machine learning–guided engineering of antibiotic resistance genes exemplifies this: A model for engineering *Escherichia coli* β-lactamase has been described and shared openly [39]. TEM-1 β-lactamase is a well-known antibiotic resistance gene that facilitates resistance of gram-negative bacteria to a range of key antibiotics, including penicillins and cephalosporins [40]. The model allows anyone with access to a computer, in theory, to generate “better-than-natural” [39] variants of TEM-1 β-lactamase. The authors claim that their work “demonstrates a generalisable and scalable paradigm for low N-protein engineering,” where low-N protein engineering refers to the minimisation of the amount of laboratory work needed to apply the approach to other protein engineering tasks. If these claims are accurate, the machine learning model in question may be applied to the design of antibiotic resistance genes to make bacteria hyperresistant, including against last-resort antibiotics. Openly shared computational methods may therefore make pathogen engineering more accessible by reducing or even removing the need for laboratory expertise and equipment.

Data may also be associated with misuse risks. The publication of experiments and genetic changes required to make avian influenza transmissible in mammals has previously raised concerns around the security risk of publishing experimental data [41]. Publicly available blueprints for particularly concerning pathogens, such as the genome of the 1918 pandemic influenza virus, feature growing potential for misuse given increasing access to viral synthesis capabilities. More powerful experimental methods mean that increasingly comprehensive datasets are generated with greater potential for misuse. For instance, in high-throughput experiments a virus may be mutated thousands of times and the effect on functions such as immune evasion or binding to human cell surface receptors recorded. Such work has been conducted on pathogens with pandemic potential including SARS-CoV-2 and influenza virus [42,43]. While these experiments are important for vaccine design, the publicly available datasets could be used by malicious actors to inform the enhancement of pandemic pathogens. Beyond the generation of datasets with greater potential for misuse, improved computational methods mean that data can be more effectively used for malicious bioengineering [38].

Publication of detailed methods, for example, for the synthesis and engineering of pandemic pathogens, may also increase the risk of accidents and misuse. Detailed protocols may lower the tacit knowledge required to perform certain procedures, making them more accessible to bad actors, inappropriately qualified personnel, or personnel working in inappropriate facilities [44]. A recent protocol detailing how to synthesise recombinant SARS-CoV-2 exemplifies this [45]. The described “reverse genetic system can be used to rapidly engineer viruses with desired mutations to study the virus in vitro and in vivo” and “enable researchers from different research backgrounds to master the use of the reverse genetic system.”

Given the misuse potential of research objects like code, datasets, and protocols, approaches for risk mitigation are needed. Across digital research objects, there appears to be a trend towards increased modularisation, i.e., sharing information in dedicated, purpose built repositories, in contrast to supplementary materials. This modularisation may allow differential access to research products according to the risk that they represent. Curated repositories with greater access control could be used that allow reuse and verification when full public disclosure of a research object is inadvisable. Such repositories are already critical for life sciences that deal with personally identifiable information. Peer reviewers could be given access during the article submission process, and subsequent access controls could be defined based on the perceived risk of the information. When information cannot be shared publicly, access controlled repositories would allow researchers to get credit for digital research object curation and creation through publication of persistent and citable metadata [46]. The Harvard Dataverse (https://dataverse.harvard.edu/) is an example of an existing repository that allows archiving of data and code with customisable restrictions and searchable metadata to facilitate discoverability. While not dedicated to protocols, the Open Science Framework does allow controlled access to all research objects that it hosts. If functionality of servers dedicated to protocols is needed, private workspaces, such as those available at protocols.io, may be suitable with adaptation.

Nonpublic clinical datasets may represent a useful model for concerning biological datasets. Participant level data often cannot be shared openly due to concerns with anonymity, so databases that regulate access are required. Clinical Practice Research Datalink (CPRD), for example, collects a range of primary healthcare data from general practices across the UK [47]. Access to data is provided through a research data governance process, which includes screening of applicants and review of a protocol. Access is only granted to bona fide researchers with trustworthy funders, and researchers must “have viable plans that maintain public and professional trust, ensure the research is of public benefit, and are methodologically robust” (https://cprd.com/Data-access). There is a thriving open science community with extensive code sharing related to analysis of such datasets, facilitated by use of standardised data formats [48]. OpenSAFELY (https://www.opensafely.org/) is another example of a project enabling open science while protecting patient confidentiality. Unlike CPRD, one of the leading influenza and coronavirus sequence sharing platforms, GISAID (www.gisaid.org), requires agreement to a Database Access Agreement, but that agreement does not have any statement regarding the purpose of the research. More comprehensive agreements may be warranted when there are concerns over data misuse. “Code only access,” where analytic code is run on datasets but researchers do not see the data directly [49,50], is another possibility.

If widespread access to code or data is desirable, an application programming interface (API; the “mechanism by which users communicate with computers, code and databases in an automated way” [51]) could be used so that certain model functions or data can be freely accessed while use for nefarious purposes is prevented. In the context of datasets, APIs have been recommended, for example, for public health bioinformatics [51]. The risk-aware rollout of the OpenAI API platform for the GPT-3 language model provides precedent. Access was initially limited while risks were assessed, and, based on the assessed risks, the API continues to limit use and imposes safety standards on third-party applications [52].

Access controls and APIs might unnecessarily interfere with scientific research while not effectively preventing misuse. In the context of personal information, processes for controlled access to the database of Genotypes and Phenotypes (dbGaP) have been criticised for being unnecessarily difficult [53]. Of particular concern are the criteria used to determine access at a global level. For example, the use of institutional track records to establish trustworthiness may conflict with ensuring equitable access to scientific findings. Difficult trade-offs will inevitably need to be made. Interdisciplinary discussion drawing on social sciences, legal, biosecurity, and life science expertise may create practical guidance for addressing these trade-offs as well as measures for evaluating success.

The use of APIs and access-controlled repositories could have benefits outside improved security. The “FAIR Guiding Principles” identify 4 principles for shared research objects: They should be findable, accessible, interoperable, and reusable for machines and people [54]. Though these principles have largely been applied to data, they are intended to apply to other digital research objects including research software [55]. APIs may increase reusability of computational models and aspects of accessibility (i.e., ability to actually use a model [55]). Code is often difficult to run due to dependencies, computing power requirements, or need for specialist skills; APIs can make it easy for anyone to use software. Access-controlled repositories may facilitate interoperability, reuse, and findability through enforcing or encouraging standards for metadata with common vocabularies and appropriate documentation; much data that is available openly is currently challenging to use and find. It is worth noting that the “A” of FAIR, accessibility, is often qualified: “as open as possible and as closed as necessary” [56]. Limiting access on the basis of security or safety concerns would not necessarily contradict this. Developing suitable repositories may therefore provide an opportunity to improve adherence to the FAIR principles and encourage their adoption across a wider range of research objects.

In the absence of appropriate sharing methods, an immediately implementable recommendation for data, code, and materials repositories is to require a declaration that the submitted information does not have dual-use potential according to a list of criteria and to reject those that do unless mitigation is in place. When data cannot be shared at all, verification and assessment of reproducibility may still be possible. For example, simulated data, a subset of the data that represents less risk, or data that has been redacted to remove concerning information could be shared. Publication of a checksum with the analysis code is a further option [57].

## Preprint publishing: A changing publication landscape offers challenges and opportunities

The use of preprints—author-formatted articles publicly deposited in a repository—in biological and medical sciences has recently increased considerably [58]. Preprints offer a faster route to publishing research than traditional journals and increase the accessibility and ability to rapidly reuse research. There are proposals for funders to mandate preprint posting [59], and several journals now act as “overlay journals,” collating and reviewing articles that have already been published as preprints [60,61]. A key difference compared to journal articles is that some preprint servers do not screen scientific articles before they are made publicly available: In one analysis, 68% provided some form of screening or moderation before the article was made public [62]. Three popular servers for biological research screen all submissions before posting, though the scope of that screening differs (Box 1).

**Box 1. Preliminary investigation into preprint policies reveals potential gaps**

We investigated the policies for screening preprints at 3 major preprint servers—bioRxiv, medRxiv, and arXiv—based on publicly available information. We read submission guidelines, frequently asked questions, and moderation policies, and searched for the server names along with terms like “dual-use” and “dangerous” to identify other relevant information.

- medRxiv submissions are screened for “material that could potentially endanger the health of individual patients or the public,” which “may include, but is not limited to, studies describing dual-use research” [63]. When launched in 2019, a cofounder of medRxiv stated that they would “almost certainly not post” studies of pathogens that could cause harm [64], and medRxiv have historically “declined work involving pathogens of pandemic potential” [65]. During the COVID-19 pandemic, however, medRxiv began accepting work on SARS-CoV-2 because “knowledge about viral variants gained from this work should be disseminated rapidly” [65].
- bioRxiv submissions “undergo a basic screening process for … material that might pose a health or biosecurity risk” [66] and “dangerous” [67] content. We were unable to find further public information on what research would be considered a biosecurity risk or dangerous. However, we have identified several papers describing viral engineering approaches posted on bioRxiv, suggesting a fairly permissive standard [68,69].
- arXiv submissions are moderated, though the explanation of the moderation process [70] does not mention dual-use, safety, security, or similar terms. Since the scope of arXiv includes “quantitative biology” [71], this may be an important shortcoming. Articles presenting data or models that were rejected from bioRxiv or medRxiv due to security concerns might be permitted at arXiv. We are aware of at least one example of a paper that was not accepted at bioRxiv due to concerns about public health later being posted on arXiv [72].

The existence of relevant wording for bioRxiv and medRxiv is promising, though the lack of mention of security or safety in arXiv may be an important gap. A more comprehensive study including more servers and details of policy implementation is needed.

Preprints may therefore remove the “gatekeeper” role that journals could play in mitigating risks from the publication of research with potential for misuse. Authors may select preprint servers that do not screen research. Unlike publishing in particular journals, there is little incentive to post to a particular preprint server, so little reason not to select one that will immediately post the article. Whether this is important depends on the role that journals play in preventing or altering publication of research with potential for misuse. There are many routes to making information available publicly over which journals have no control, such as personal websites, news articles, or conference presentations. However, there are examples where journals and editors have been important in evaluating risks from publication: For example, in 2014, information was redacted from 2 manuscripts about gene sequences of a novel *Clostridium botulinum* toxin following consultation between editors, authors, and branches of the US government [73,74]. Some journals also request that reviewers help to identify dual-use risks [73]. Preprints may therefore increase the probability that dangerous methods or results are described publicly. Preprints challenge any model relying on review by journals at publication [18], emphasising the need for oversight at other stages in the research life cycle, such as during design and funding. The need to consider preprints in the context of research with dual-use potential has been expressed previously [75].

As recommended for code, data, and materials, preprint servers could require a declaration that the posted research does not present dual-use potential and reject posting of articles that do not provide this. There are few prominent preprint servers in comparison to the number of journals, which may represent an opportunity for coordination. Relatively few parties would need to agree on a policy for it to be implemented universally, or at least across all of the major servers (such as bioRxiv, medRxiv, arXiv, OSF preprints, and preprints.org). If an article is flagged by at least one server as potentially concerning, other servers could agree not to post that article until it was appropriately peer reviewed. However, authors must be incentivised to post to those preprint servers with appropriate review processes, and funders, institutions, and possibly journals, rather than researchers, will likely need to encourage this. Further research into the role that preprints play in influencing biosafety and biosecurity risks, policies currently in place, and proposals to mitigate those risks, would be valuable.

## Preregistration: An opportunity for dual-use oversight

Preregistration means archiving a time-stamped protocol that can be referred back to once a project is completed. This protocol is generally made public. Study registries, such as clinicaltrials.gov for clinical trials or PROSPERO for systematic reviews, represent a minimal form of preregistration where details of the study design and study outcomes are provided. In a stronger form, preregistration involves providing a detailed plan for the conduct and analysis of a study, including possibly the analytic code. Such plans are often registered on the Open Science Framework or As Predicted. Preregistration aims to limit duplication and selective reporting through disclosure of research plans [76] and reduce the prevalence of questionable research practices by clearly distinguishing planned and exploratory analyses [77].

It seems likely that greater consideration of the research before it is started, as encouraged by preregistration, could help to mitigate misuse risks. Currently, biosecurity risk assessment and management is not consistently conducted at any stage throughout the research lifecycle; preregistration could encourage greater consideration of risks at an early stage. Submission platforms could ask researchers to reflect on the dual-use potential of their work. In certain high-risk fields, platforms could request that details of hazard assessment be provided, which could be incentivised by journals requesting evidence of such assessments on publication. A safety and security form is required as part of the International Genetically Engineered Machine (iGEM) competition [78], which may be a useful model. In cases where researchers are unsure or do have concerns, they could be directed to an expert or relevant resources.

Registered reports are a type of preregistration and journal article that may present an opportunity for oversight [79]. In registered reports, the introduction and methods (the stage one submission) are peer-reviewed before the work begins. If the stage one submission is accepted, the authors are guaranteed publication of the complete article (the stage two submission) regardless of the results, provided that they follow the proposed methods and the interpretation of the results is reasonable. Peer review at an earlier stage in the research life cycle represents a clear opportunity. When stage one submissions are received that could present biosecurity or biosafety risks, editors could assign biosecurity experts to risk-assess the article, in parallel with the traditional scientific peer review. This is similar to how registered reports currently operate, with specialists in different areas relevant to the manuscript (e.g., statistics, particular methods) reviewing submissions. A biosecurity report could detail recommendations on how the research could be conducted and reported to minimise risk, or, if necessary, advise against conduct altogether. In cases where review identifies risks, it remains an open question whether review reports should be made openly available. Sharing may encourage responsible discourse, but some arguments against conducting research may themselves present risk by highlighting how research could be misused.

The format of registered reports and preregistrations is currently focussed on hypothesis-testing, confirmatory research. In general, concerning biological research is more likely to be exploratory, involving discovery or development of tools, and without methods and aims that can be defined adequately in advance. However, there are proposals in the open science community to adapt preregistrations to be suitable for exploratory work [80]. If implemented, early collaboration with biosecurity experts would be advantageous in ensuring that risk of misuse is one of the criteria considered. Proactively encouraging early trials of any new format of advisory peer review in areas of perceived high risk, such as synthetic mammalian virology, as suggested elsewhere [20], could be beneficial. Addressing dual-use risks at the early stages of the research lifecycle may be more effective than suppressing the dissemination of dangerous insights after work is completed. Therefore, interventions aimed at encouraging review at the conception of research seem particularly promising.

## The way forward

We highlight several opportunities for reducing risks from research with dual-use potential. First, increased modularisation of research may facilitate differential sharing of research outputs depending on the risk they represent. There is a need to evaluate the suitability of existing tools that are used for sharing in terms of usability and security. To encourage the maximal adoption of tools that facilitate restricted access when needed, they must be as simple as possible to use from the perspective of both the researchers depositing materials and later users of those materials. They must also be secure. If existing tools are not suitable, new platforms may need to be developed. In either case, encouraging and monitoring the adoption of security-friendly sharing practices will be essential. Second, preprints may remove any gatekeeper role that journals play, as evidenced by the lack of screening by some preprint servers, emphasising the need for oversight throughout the research lifecycle rather than solely at the publication stage. Finally, preregistration and registered reports may encourage greater consideration of dual-use potential early in the research process. Existing preregistration formats will require adaptation to be suitable for this purpose. There is a need for guidance and input from individuals or organisations with experience in assessing research with dual-use potential to guide and pilot those adaptations in relevant communities.

The concerns and proposals discussed here may be relevant to a range of research areas. Aspects of vaccine research, gene therapy research, and cancer therapeutics development may be associated with risks [81]. Considerable discussion has taken place in the artificial intelligence community about the potential for misuse of published code, such as in the context of deepfake videos for blackmail [82]. Studies that model terrorist scenarios are available, which might assist bad actors in predicting the impact of attacks. It is difficult to identify and anticipate all concerning research areas and formal regulation cannot keep pace [78]. It is therefore important to consider the possibility of research misuse when developing and implementing new open science tools and initiatives for general use.

Incentives for open science require careful consideration. Many have been proposed, including changing hiring practices to support open data, open materials and preregistration [83–85], open science leaderboards [86], journal scores based on transparency [87], badges acknowledging open science on published papers [88], and assessment of open science practices by funders [89]. These incentives must allow limited disclosure when it is justified on the basis of safety or security concerns. Open data badges, for example, are available “if sensitive, personal, data are available only from an approved third party” [90] but not explicitly when the data exhibits safety or security risk. Researchers must not be penalised for responsible disclosure or incentivised to disclose irresponsibly.

Any proposal to allow researchers to reduce public sharing could be exploited by those unwilling to invest the effort that open science requires. For example, researchers who have inadequately documented or fabricated data might invent security concerns. Alternatives to public sharing must therefore include appropriate verification, perhaps through peer review, to ensure that they are available for reuse when appropriate. Since this may increase the burden on reviewers and editors, there may be a need for individuals or organisations with appropriate expertise who are willing to and capable of providing this service. Funders interested in biosecurity and biosafety could support this.

Funders and institutions have an important role to play in improving biosecurity. Storing data in approved platforms, posting only to preprint servers with adequate review processes, and preregistration of research with high potential for misuse, for example, could be mandated or encouraged by funders or institutional oversight groups. While solutions are developed and implemented, clear policies should be in place for the communication of research outputs that involve safety or security risks. Common to much discussion in this paper is the need for input from experts in risks of biological research. As these risks appear to be relatively neglected, this may be a key bottleneck in developing and implementing changes. Greater investment in expertise related to biosecurity and biosafety will likely be important for realisation of any proposals involving peer review for risk assessment or mitigation purposes. More generally, consideration of downside risk of both open science and biological science appears to be neglected in comparison to its plausible magnitude. Education and outreach may help to increase awareness among relevant stakeholders.

## Conclusions

Open science practices may in many cases contribute positively to our ability to deal with biological threats. However, given the many concerning examples of biosafety and biosecurity incidents, the potential threat posed by the increasing accessibility and usability of scientific research to all actors cannot be ignored. Increased sharing of code, data, and materials in particular are concerning in some cases. There is an urgent need to address the inadvertent risks associated with certain open science practices and encourage responsible sharing and access. For preprints, the lack of screening in some cases challenges strategies relying on assessment of dual-use potential at the publication stage, but interventions may be possible and should be explored. Preregistration is a useful model that could encourage risk assessment and advisory peer review of research with dual-use potential earlier in the research lifecycle. In general, there is a need for ongoing, critical evaluation of incumbent and changing scientific practices, and consideration of the risks that such practices represent.

## Abbreviations

API: application programming interface
CPRD: Clinical Practice Research Datalink
dbGaP: database of Genotypes and Phenotypes
DURC: dual-use research of concern
iGEM: International Genetically Engineered Machine

## References

[1] Wallach JD, Boyack KW, Ioannidis JPA. Reproducible research practices, transparency, and open access data in the biomedical literature, 2015–2017. PLoS Biol. 2018;16:e2006930. doi: 10.1371/journal.pbio.2006930 30457984 PMC6245499

[2] Serghiou S, Contopoulos-Ioannidis DG, Boyack KW, Riedel N, Wallach JD, Ioannidis JPA. Assessment of transparency indicators across the biomedical literature: How open is open? PLoS Biol. 2021;19:e3001107. doi: 10.1371/journal.pbio.3001107 33647013 PMC7951980

[3] Beeckman DSA, Rüdelsheim P. Biosafety and Biosecurity in Containment: A Regulatory Overview. Front Bioeng Biotechnol. 2020;8:650. doi: 10.3389/fbioe.2020.00650 32719780 PMC7348994

[4] Jackson SS, Sumner LE, Garnier CH, Basham C, Sun LT, Simone PL, et al. The accelerating pace of biotech democratization. Nat Biotechnol. 2019;37:1403–8. doi: 10.1038/s41587-019-0339-0 31796931

[5] Manheim D, Lewis G. High-risk human-caused pathogen exposure events from 1975–2016. F1000Res. 2021. doi: 10.12688/f1000research.55114.1 PMC9274012 35903214

[6] Palese P. Influenza: old and new threats. Nat Med. 2004;10:S82–7. doi: 10.1038/nm1141 15577936

[7] Enemark C. Biosecurity Dilemmas: Dreaded Diseases, Ethical Responses, and the Health of Nations. Georgetown University Press; 2017. Available from: https://www.jstor.org/stable/j.ctt1kk672v.

[8] Sosin DM. Review of Department of Defense Anthrax. Shipments. 2015;13.

[9] Klytz L. Human error in high-biocontainment labs: a likely pandemic threat. Bull At Sci [Internet]. 25 Feb 2019 [cited 15 Aug 2021]. Available from: https://thebulletin.org/2019/02/human-error-in-high-biocontainment-labs-a-likely-pandemic-threat/.

[10] Demaneuf G. The Good, the Bad and the Ugly: a review of SARS Lab Escapes. Zenodo. 2020 Nov 27. doi: 10.5281/zenodo.5091301

[11] Gilsdorf JR, Zilinskas RA. New Considerations in Infectious Disease Outbreaks: The Threat of Genetically Modified Microbes. Clin Infect Dis. 2005;40:1160–5. doi: 10.1086/428843 15791517

[12] Bush LM, Perez MT. The Anthrax Attacks 10 Years Later. Ann Intern Med. 2012;156:41–4. doi: 10.7326/0003-4819-155-12-201112200-00373 21969275

[13] Dual-Use Research | NIH Office of Intramural Research. [cited 28 Jun 2021]. Available from: https://oir.nih.gov/sourcebook/ethical-conduct/special-research-considerations/dual-use-research.

[14] Agenda 2024 GHS. APP3 Statement on Biosecurity and Biosafety During the COVID-19 Pandemic. Global Health Security Agenda [Internet]. 30 Jul 2020 [cited 16 Aug 2021]. Available from: https://ghsagenda.org/2020/07/30/app3-statement-on-biosecurity-and-biosafety-during-the-covid-19-pandemic/.

[15] United States Government Policy for Oversight of Life Sciences Dual Use Research of Concern. 2012.

[16] United States Government Policy for Institutional Oversight of Life Sciences Dual Use Research of Concern. 2014. Available from: https://www.phe.gov/s3/dualuse/documents/oversight-durc.pdf.

[17] Morland H. Born secret. Cardozo L Rev. 2004;26:1401.

[18] Musunuri S, Sandbrink J, Monrad J, Palmer M, Koblentz G. Rapid proliferation of pandemic research: implications for dual-use risks. mBio 2021;12. doi: 10.1128/mBio.01864-21 34663091 PMC8524337

[19] Lewis G, Millett P, Sandberg A, Snyder-Beattie A, Gronvall G. Information Hazards in Biotechnology. Risk Anal. 2019;39:975–81. doi: 10.1111/risa.13235 30419157 PMC6519142

[20] Esvelt KM. Inoculating science against potential pandemics and information hazards. PLoS Pathog. 2018;14:e1007286. doi: 10.1371/journal.ppat.1007286 30286188 PMC6171951

[21] Nosek BA, Alter G, Banks GC, Borsboom D, Bowman SD, Breckler SJ, et al. Promoting an open research culture. Science. 2015;348:1422–5. doi: 10.1126/science.aab2374 26113702 PMC4550299

[22] Miguel E, Camerer C, Casey K, Cohen J, Esterling KM, Gerber A, et al. Promoting Transparency in Social Science Research. Science. 2014;343:30–1. doi: 10.1126/science.1245317 24385620 PMC4103621

[23] Munafò MR, Nosek BA, Bishop DVM, Button KS, Chambers CD, Percie du Sert N, et al. A manifesto for reproducible science. Nat Hum Behav. 2017;1:0021. doi: 10.1038/s41562-016-0021 33954258 PMC7610724

[24] Baker M. 1,500 scientists lift the lid on reproducibility. Nature. 2016;533:452–4. doi: 10.1038/533452a 27225100

[25] Goodman SN, Fanelli D, Ioannidis JPA. What does research reproducibility mean? Sci Transl Med. 2016;8:341ps12-341ps12. doi: 10.1126/scitranslmed.aaf5027 27252173

[26] Levin N, Leonelli S, Weckowska D, Castle D, Dupré J. How Do Scientists Define Openness? Exploring the Relationship Between Open Science Policies and Research Practice. Bull Sci Technol Soc. 2016;36:128–41. doi: 10.1177/0270467616668760 27807390 PMC5066505

[27] Vicente-Saez R, Martinez-Fuentes C. Open Science now: A systematic literature review for an integrated definition. J Bus Res. 2018;88:428–36. doi: 10.1016/j.jbusres.2017.12.043

[28] Allen C, Mehler DMA. Open science challenges, benefits and tips in early career and beyond. PLoS Biol. 2019;17:e3000246. doi: 10.1371/journal.pbio.3000246 31042704 PMC6513108

[29] Fraser N, Brierley L, Dey G, Polka JK, Pálfy M, Nanni F, et al. The evolving role of preprints in the dissemination of COVID-19 research and their impact on the science communication landscape. PLoS Biol. 2021;19:e3000959. doi: 10.1371/journal.pbio.3000959 33798194 PMC8046348

[30] Information NC for B, Pike USNL of M 8600 R, MD B, Usa 20894. Responsible Life Sciences Research for Global Health Security. World Health Organization; 2010. Available from: https://www.ncbi.nlm.nih.gov/books/NBK305040/. 26180872

[31] Atlas RM, Dando M. The dual-use dilemma for the life sciences: perspectives, conundrums, and global solutions. Biosecur Bioterror. 2006;4:276–86. doi: 10.1089/bsp.2006.4.276 16999588

[32] Baker M. Why scientists must share their research code. Nature. 2016 [cited 17 Aug 2021]. doi: 10.1038/nature.2016.20504

[33] Goldacre B, Morton CE, DeVito NJ. Why researchers should share their analytic code. BMJ. 2019;367:l6365. doi: 10.1136/bmj.l6365 31753846

[34] Scientific Data recommended repositories. figshare; 2019. doi: 10.6084/m9.figshare.1434640.v16

[35] Ihle M, Bishop D, Fortunato L. Open research at Oxford survey. 2021 [cited 1 Nov 2021]. doi: 10.5281/zenodo.4437067

[36] Bewley KR, Coombes NS, Gagnon L, McInroy L, Baker N, Shaik I, et al. Quantification of SARS-CoV-2 neutralizing antibody by wild-type plaque reduction neutralization, microneutralization and pseudotyped virus neutralization assays. Nat Protoc. 2021;16:3114–40. doi: 10.1038/s41596-021-00536-y 33893470

[37] Carlson CJ, Farrell MJ, Grange Z, Han BA, Mollentze N, Phelan AL, et al. The future of zoonotic risk prediction. Philos Trans R Soc Lond B Biol Sci. 2021;376. doi: 10.1098/rstb.2020.0358 34538140 PMC8450624

[38] Sandbrink JB, Alley EC, Watson MC, Koblentz GD, Esvelt KM. Insidious Insights: Implications of viral vector engineering for pathogen enhancement. Gene Ther. 2022:1–4. doi: 10.1038/s41434-021-00312-3 35264741 PMC10191845

[39] Biswas S, Khimulya G, Alley EC, Esvelt KM, Church GM. Low-N protein engineering with data-efficient deep learning. Nat Methods. 2021;18:389–96. doi: 10.1038/s41592-021-01100-y 33828272

[40] Salverda MLM, De Visser JAGM, Barlow M. Natural evolution of TEM-1 β-lactamase: experimental reconstruction and clinical relevance. FEMS Microbiol Rev. 2010;34:1015–36. doi: 10.1111/j.1574-6976.2010.00222.x 20412308

[41] Berns KI, Casadevall A, Cohen ML, Ehrlich SA, Enquist LW, Fitch JP, et al. Adaptations of Avian Flu Virus Are a Cause for Concern. Science. 2012;335:660–1. doi: 10.1126/science.1217994 22294736

[42] Lee JM, Eguia R, Zost SJ, Choudhary S, Wilson PC, Bedford T, et al. Mapping person-to-person variation in viral mutations that escape polyclonal serum targeting influenza hemagglutinin. Lipsitch M, Kirkegaard K, Lipsitch M, editors. Elife. 2019;8:e49324. doi: 10.7554/eLife.49324 31452511 PMC6711711

[43] Starr TN, Greaney AJ, Hilton SK, Crawford KHD, Navarro MJ, Bowen JE, et al. Deep mutational scanning of SARS-CoV-2 receptor binding domain reveals constraints on folding and ACE2 binding. 2020 Jun p. 2020.06.17.157982. doi: 10.1101/2020.06.17.157982 32841599 PMC7418704

[44] Pannu J, Sandbrink JB, Watson M, Palmer MJ, Relman DA. Protocols and risks: when less is more. Nat Protoc. 2021:1–2. doi: 10.1038/s41596-021-00655-6 34873329

[45] Xie X, Lokugamage KG, Zhang X, Vu MN, Muruato AE, Menachery VD, et al. Engineering SARS-CoV-2 using a reverse genetic system. Nat Protoc. 2021;16:1761–84. doi: 10.1038/s41596-021-00491-8 33514944 PMC8168523

[46] Hrynaszkiewicz I, Khodiyar V, Hufton AL, Sansone S-A. Publishing descriptions of non-public clinical datasets: proposed guidance for researchers, repositories, editors and funding organisations. Res Integr Peer Rev. 2016;1:6. doi: 10.1186/s41073-016-0015-6 29451541 PMC5793987

[47] Herrett E, Gallagher AM, Bhaskaran K, Forbes H, Mathur R, van Staa T, et al. Data Resource Profile: Clinical Practice Research Datalink (CPRD). Int J Epidemiol. 2015;44:827–36. doi: 10.1093/ije/dyv098 26050254 PMC4521131

[48] van Bochove K. Chapter 3 Open Science | The Book of OHDSI. Available from: https://ohdsi.github.io/TheBookOfOhdsi/.

[49] Dayan I, Roth HR, Zhong A, Harouni A, Gentili A, Abidin AZ, et al. Federated learning for predicting clinical outcomes in patients with COVID-19. Nat Med. 2021:1–9. doi: 10.1038/s41591-020-01213-5 34526699 PMC9157510

[50] Williamson EJ, Walker AJ, Bhaskaran K, Bacon S, Bates C, Morton CE, et al. Factors associated with COVID-19-related death using OpenSAFELY. Nature. 2020;584:430–6. doi: 10.1038/s41586-020-2521-4 32640463 PMC7611074

[51] Black A, MacCannell DR, Sibley TR, Bedford T. Ten recommendations for supporting open pathogen genomic analysis in public health. Nat Med. 2020;26:832–41. doi: 10.1038/s41591-020-0935-z 32528156 PMC7363500

[52] OpenAI API. In: OpenAI [Internet]. 11 Jun 2020 [cited 30 Aug 2021]. Available from: https://openai.com/blog/openai-api/.

[53] Powell K. The broken promise that undermines human genome research. Nature. 2021;590:198–201. doi: 10.1038/d41586-021-00331-5 33568833

[54] Wilkinson MD, Dumontier M, IjJ A, Appleton G, Axton M, Baak A, et al. The FAIR Guiding Principles for scientific data management and stewardship. Sci Data. 2016;3:160018. doi: 10.1038/sdata.2016.18 26978244 PMC4792175

[55] Lamprecht A-L, Garcia L, Kuzak M, Martinez C, Arcila R, Martin Del Pico E, et al. Towards FAIR principles for research software. Data Science. 2020;3:37–59. doi: 10.3233/DS-190026

[56] Landi A, Thompson M, Giannuzzi V, Bonifazi F, Labastida I, da Silva Santos LOB, et al. The “A” of FAIR–As Open as Possible, as Closed as Necessary. Data Intelligence. 2020;2:47–55. doi: 10.1162/dint_a_00027

[57] Van Lissa CJ, Brandmaier AM, Brinkman L, Lamprecht A-L, Peikert A, Struiksma ME, et al. WORCS: A workflow for open reproducible code in science. Data Science. 2021;4:29–49. doi: 10.3233/DS-210031

[58] Preprint summary metrics. [cited 16 Aug 2021]. Available from: https://rxivist.org/stats.

[59] Sever R, Eisen M, Inglis J. Plan U: Universal access to scientific and medical research via funder preprint mandates. PLoS Biol. 2019;17:e3000273. doi: 10.1371/journal.pbio.3000273 31163026 PMC6548351

[60] Eisen MB, Akhmanova A, Behrens TE, Harper DM, Weigel D, Zaidi M. Implementing a “publish, then review” model of publishing. Elife. 2020;9:e64910. doi: 10.7554/eLife.64910 33258772 PMC7710353

[61] Eysenbach G. Celebrating 20 Years of Open Access and Innovation at JMIR Publications. J Med Internet Res. 2019;21:e17578. doi: 10.2196/17578 31868653 PMC6945123

[62] Malicki M, Jeroncic A, ter Riet G, Bouter LM, Ioannidis JPA, Goodman SN, et al. Preprint Servers’ Policies, Submission Requirements, and Transparency in Reporting and Research Integrity Recommendations. JAMA. 2020;324:1901. doi: 10.1001/jama.2020.17195 33170231 PMC7656281

[63] medRxiv Freqeuently Asked Questions. [cited 17 Nov 2021]. Available from: https://www.medrxiv.org/about/FAQ.

[64] Kaiser J. Medical preprint server debuts. Science. 2019:5. doi: 10.1126/science.aax5052 30948528

[65] Sever R, Inglis J, Bloom T, Rawlinson C, Krumholz H, Ross J. Pandemic preprints—a duty of responsible stewardship. BMJ [Internet]. 27 Apr 2021 [cited 17 Nov 2021]. Available from: https://blogs.bmj.com/bmj/2021/04/27/pandemic-preprints-a-duty-of-responsible-stewardship/.

[66] bioRxiv Submission Guide. [cited 17 Nov 2021]. Available from: https://www.biorxiv.org/submit-a-manuscript.

[67] bioRxiv Frequently Asked Questions. [cited 17 Nov 2021]. Available from: https://www.biorxiv.org/about/FAQ.

[68] Ye C, Chiem K, Park J-G, Oladunni F, Platt RN, Anderson T, et al. Rescue of SARS-CoV-2 from a single bacterial artificial chromosome. bioRxiv; 2020. p. 2020.07.22.216358. doi: 10.1101/2020.07.22.216358 32978313 PMC7520601

[69] Thao TTN, Labroussaa F, Ebert N, V’kovski P, Stalder H, Portmann J, et al. Rapid reconstruction of SARS-CoV-2 using a synthetic genomics platform. bioRxiv; 2020. p. 2020.02.21.959817. doi: 10.1101/2020.02.21.959817 32365353

[70] arXiv moderation | arXiv e-print repository. [cited 17 Nov 2021]. Available from: https://arxiv.org/help/moderation#what-policies.

[71] arXivorg e-Print archive. [cited 17 Nov 2021]. Available from: https://arxiv.org/.

[72] Kwon D. How swamped preprint servers are blocking bad coronavirus research. Nature. 2020;581:130–1. doi: 10.1038/d41586-020-01394-6 32382120

[73] Hooper DC, Hirsch MS. Novel Clostridium botulinum Toxin and Dual Use Research of Concern Issues. J Infect Dis. 2014;209:167–7. doi: 10.1093/infdis/jit528 24106293 PMC3873790

[74] Relman DA. “Inconvenient Truths” in the Pursuit of Scientific Knowledge and Public Health. J Infect Dis. 2014;209:170–2. doi: 10.1093/infdis/jit529 24106297 PMC3873791

[75] Schloss PD. Preprinting Microbiology. mBio. 2017;8:e00438–17. doi: 10.1128/mBio.00438-17 28536284 PMC5442452

[76] ICMJE | Recommendations | Clinical Trials. [cited 17 Jun 2020]. Available from: http://www.icmje.org/recommendations/browse/publishing-and-editorial-issues/clinical-trial-registration.html.

[77] Nosek BA, Ebersole CR, DeHaven AC, Mellor DT. The preregistration revolution. Proc Natl Acad Sci U S A. 2018;115:2600–6. doi: 10.1073/pnas.1708274114 29531091 PMC5856500

[78] Millett P, Alexanian T. Implementing adaptive risk management for synthetic biology: Lessons from iGEM’s safety and security programme. Eng Biol. n/a. doi: 10.1049/enb2.12012 PMC9996700 36968257

[79] Warmbrod KL, Montague MG, Gronvall GK. COVID-19 and the gain of function debates. EMBO Rep. 2021;22:e53739. doi: 10.15252/embr.202153739 34477287 PMC8490979

[80] Dirnagl U. Preregistration of exploratory research: Learning from the golden age of discovery. PLoS Biol. 2020;18:e3000690. doi: 10.1371/journal.pbio.3000690 32214315 PMC7098547

[81] Sandbrink JB, Koblentz GD. Biosecurity risks associated with vaccine platform technologies. Vaccine. 2021;S0264-410X(21):00171–7. doi: 10.1016/j.vaccine.2021.02.023 33640142 PMC7904460

[82] Shevlane T, Dafoe A. The Offense-Defense Balance of Scientific Knowledge: Does Publishing AI Research Reduce Misuse? Proceedings of the AAAI/ACM Conference on AI, Ethics, and Society. New York, NY, USA: Association for Computing Machinery; 2020. p. 173–179. doi: 10.1145/3375627.3375815

[83] Schönbrodt F. Changing hiring practices towards research transparency: The first open science statement in a professorship advertisement. Nicebread [Internet]. 6 Jan 2016 [cited 2 Jul 2021]. Available from: https://www.nicebread.de/open-science-hiring-practices/.

[84] Dirnagl Ulrich. If you are applying for a professorship at the Charite you now need to tell us about your contributions to your scientific field, open science, team science, interactions with stakeholders. Past and future plans. As a structured narrative. https://t.co/lm3aXBGSE0. In: @dirnagl [Internet]. 4 Mar 2018 [cited 2 Jul 2021]. Available from: https://twitter.com/dirnagl/status/970227847943114752.

[85] Buck S. Beware performative reproducibility. Nature. 2021;595:151–1. doi: 10.1038/d41586-021-01824-z 34230657

[86] Transparency Audits for Science—Curate Science. [cited 2 Jul 2021]. Available from: https://curatescience.org.

[87] Woolston C. TOP Factor rates journals on transparency, openness. 2020 [cited 2 Jul 2021]. Available from: https://www.natureindex.com/news-blog/top-factor-rates-journals-on-transparency-openness.

[88] Kidwell MC, Lazarević LB, Baranski E, Hardwicke TE, Piechowski S, Falkenberg L-S, et al. Badges to Acknowledge Open Practices: A Simple, Low-Cost, Effective Method for Increasing Transparency. PLoS Biol. 2016;14:e1002456. doi: 10.1371/journal.pbio.1002456 27171007 PMC4865119

[89] de Jonge H, Cruz M, Holst S. Funders need to credit open science. Nature. 2021;599:372–2. doi: 10.1038/d41586-021-03418-1 34785800

[90] Approved Protected Access Repositories. Badges to Acknowledge Open Practices. 2020 [cited 2 Jul 2021]. Available from: https://osf.io/tvyxz/wiki/8.%20Approved%20Protected%20Access%20Repositories/.

